# Voltage slope guided learning in spiking neural networks

**DOI:** 10.3389/fnins.2022.1012964

**Published:** 2022-11-10

**Authors:** Lvhui Hu, Xin Liao

**Affiliations:** ^1^School of Intelligent Medicine, Chengdu University of Traditional Chinese Medicine, Chengdu, China; ^2^Information Center, Hospital of Chengdu University of Traditional Chinese Medicine, Chengdu, China

**Keywords:** spiking neural networks, spiking neurons, aggregate-label learning, temporal credit-assignment, synaptic adjustment

## Abstract

A thorny problem in machine learning is how to extract useful clues related to delayed feedback signals from the clutter of input activity, known as the temporal credit-assignment problem. The aggregate-label learning algorithms make an explicit representation of this problem by training spiking neurons to assign the aggregate feedback signal to potentially effective clues. However, earlier aggregate-label learning algorithms suffered from inefficiencies due to the large amount of computation, while recent algorithms that have solved this problem may fail to learn due to the inability to find adjustment points. Therefore, we propose a membrane voltage slope guided algorithm (VSG) to further cope with this limitation. Direct dependence on the membrane voltage when finding the key point of weight adjustment makes VSG avoid intensive calculation, but more importantly, the membrane voltage that always exists makes it impossible to lose the adjustment point. Experimental results show that the proposed algorithm can correlate delayed feedback signals with the effective clues embedded in background spiking activity, and also achieves excellent performance on real medical classification datasets and speech classification datasets. The superior performance makes it a meaningful reference for aggregate-label learning on spiking neural networks.

## 1. Introduction

The birth and development of artificial intelligence are deeply inspired by the sophisticated biological brain, such as the striking deep learning represented by the artificial neural network (ANNs), which has attracted considerable attention in the past decade (LeCun et al., 2015). ANNs highly abstract biological neurons, and obtains the analog outputs by the weighted sum of the analog inputs through activation function. This conversion process is somewhat consistent with the biological spiking process, and the analog inputs and outputs are also regarded as equivalent to the firing rates of biological neurons (Rueckauer et al., 2017). However, ANNs still lack biological realism compared to physiological neural networks that utilize binary spikes for information transfer (Bengio et al., 2015).

Then, spiking neural networks (SNNs) offer a new computing paradigm with theoretical advantages in computational efficiency and power consumption due to the adoption of the binary spiking mechanism. However, these advantages have not been fully exploited, and the results are far from achieving the desired impact. One of the major reasons is the lack of efficient learning algorithms, so research on SNN algorithms remains attractive. Nevertheless, many valuable works have emerged. Among them, depending on the presence of additional teaching signals, existing SNN algorithms can be roughly divided into supervised and unsupervised.

Neurophysiological studies have shown that the long-term potentiation (LTP) and depression (LTD) of synaptic transmission are ubiquitous phenomena existing in almost every excitatory synapse in the mammalian brain (Malenka and Bear, 2004). Spike-timing dependent plasticity (STDP) rule (Bi and Poo, 1998), which combines these two phenomena, becomes a feasible unsupervised learning rule benefiting by its definite biological basis. Then STDP intrigues the research of local learning rules that imitate the neuroscience mechanisms (Masquelier et al., 2007; Diehl and Matthew, 2015; Tavanaei and Maida, 2017a). For example, STDP rules have been applied to an SNN architecture that simulates visual function to promote neurons show the selectivity of orientation and disparity (Barbier et al., 2021), to shallow convolutional SNNs to realize near-real-time processing of events collected from neuromorphic vision sensors (She and Mukhopadhyay, 2021), and to weight-quantized SNNs to complete online learning (Hu et al., 2021), etc. In addition, variants of STDP have also been embedded into Inception-like SNNs for highly parallel feature extraction (Meng et al., 2021) or ensemble convolutional SNNs for object recognition (Fu and Dong, 2021). This biologically inspired learning do not require regulatory signals and is easy to execute, making it attractive to hardware implementation of emerging memory devices (Burr et al., 2016; Zhou et al., 2022). However, such local learning rules are more suitable for small-scale pattern recognition tasks, and it is difficult for them to be directly applied in complex tasks due to the lack of global information related to convergence for large models (Mozafari et al., 2018).

On the other hand, there is also documented evidence supporting the existence of instruction-based learning in the central nervous system (Knudsen, 1994; Thach, 1996). Over the years, a growing number of supervised learning algorithms of SNN have been proposed (Ponulak and Kasiński, 2010; Florian, 2012; Mohemmed et al., 2012; Xu et al., 2013b; Memmesheimer et al., 2014; Zhang et al., 2018a,b, 2019; Luo et al., 2022), and some of them obtained comparable accuracies to that of ANNs in large-scale applications. SpikeProp (Bohte et al., 2000) is a classical supervised learning method of SNNs, which is derived from the gradient descent algorithm of ANNs. While the application of this algorithm is limited by the fact that each neuron can only fire once, so the multi-spike version of it are proposed to improve performance (Ghosh-Dastidar and Adeli, 2009; Xu et al., 2013a). As for the critical dilemma of non-differentiable discrete spikes in SNNs, Spikeprop uses a linear assumption of membrane potential at these time instants to bypass it. The other way proposed in SLAYER (Shrestha and Orchard, 2018) to handle it is to replace the derivatives of these non-differentiable moments with approximate functions, SuperSpike (Zenke and Ganguli, 2018) algorithm uses the surrogate gradients, and DSR (Meng et al., 2022) uses gradients of sub-differentiable mappings. These algorithms and some others (Wu et al., 2018c, 2019) almost all follow the idea of back-propagation through time (BPTT), which makes full use of information on both time and space scales, but it also means quite a bit of computing and storage requirements.

Beyond these, there are some situations where the guidance signals are ambiguous. For example, animal survival behavior to identify whether small clues in the environment represent danger or opportunity involves detecting relationships between multiple clues and ambiguous long-delayed feedback signals. Multi-Spike Tempotron (MST) (Gütig, 2016), an aggregate-label learning algorithm, is proposed to train a detector to automatically respond wherever a valid clue appears, given only the number of desired spikes. It uses the distance between the true threshold and the a critical threshold (under which a specific number of spikes can be fired) as the error signal for weight adjustment, enabling it to obtain robust and powerful learning capabilities. Then TDP1 (Yu et al., 2018) is proposed to simplify the iteration calculation in MST and improve the learning efficiency. However, they are still computationally expensive due to the need to calculate the critical threshold. Therefore, MPD-AL (Zhang et al., 2019) directly adjusts the weight from the membrane voltage, which greatly reduces the computing requirements. However, the disadvantage of this method is that there is a possibility that the tunable point cannot be found.

Inspired by MPD-AL, we propose an voltage slope-guided algorithm (VSG). When the number of spikes emitted by the output neuron is not equal to its desired spike number, an appropriate point is selected to adjust the weight according to the slope of the membrane voltage, so that the neuron can emit more spikes or remove redundant spikes. The proposed method avoids the dilemma of failing to find the adjustment points, and does not need iterative calculation to find the critical threshold. The comparative experiments with MPD-AL, MST, and TDP1 verify its superiority, and the classification results on realistic datasets further proves its practical performance.

The rest of the article is organized as follows: In Section 2, we introduce the proposed algorithm and compare it with several other algorithms. In Section 3, we conduct a series of experiments to verify the performance of the algorithm. Finally, the algorithm is analyzed and discussed in Section 4.

## 2. Neuron model and learning algorithm

In this section, the neuron model employed will be first introduced, followed by the proposed VSG algorithm, and finally this algorithm will be compared with its counterparts, highlighting their differences.

### 2.1. Neuron model

The leaky integrate-and-fire neuron model (Maass and Bishop, 1999; Gütig, 2016) is one of the most widely used spiking neuron models, benefiting from its computational simplicity and modest biological reliability. So we also adopt it in this article.

**Figure 1:**
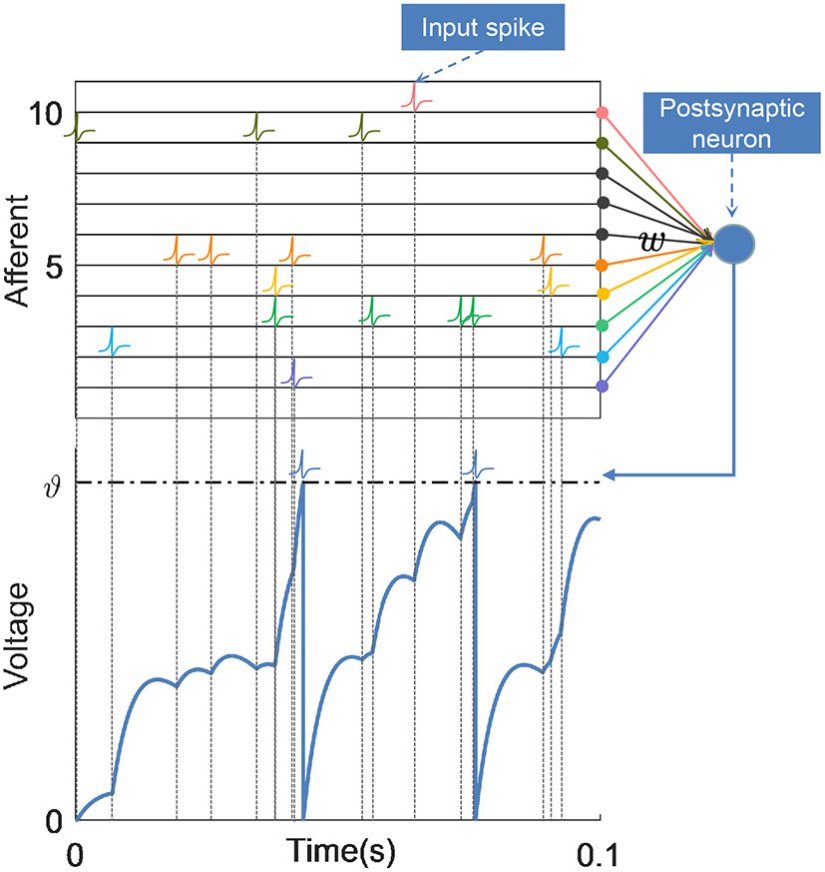
Schematic of neuronal structure and membrane voltage dynamics. The postsynaptic neuron integrates spikes from afferent neurons over time, and each incoming spike contributes to the membrane voltage together with the corresponding synaptic weights. When the membrane voltage of the postsynaptic neuron crosses the firing threshold ϑ, it emits a spike and the voltage is reset.

The postsynaptic neuron receives spikes transmitted from its *N* presynaptic neurons through synapses, which induce postsynaptic potentials (PSPs) on the postsynaptic neuron, resulting in changes in its membrane voltage *V*(*t*), as shown in Figure 1. Thus, the membrane voltage of the postsynaptic neuron gradually rises from the resting state *V*_*rest*_ = 0. When the membrane voltage crosses the threshold ϑ, the neuron fires a spike, and the membrane voltage quickly resets to the resting potential, then it enters a refractory period. This process can be expressed as:


(1) $$\begin{array}{l} V\left(t\right)=V_{rest}+\overset{N}{\underset{i=1}{\sum }}w_{i}\underset{t_{i}^{j}<t}{\sum }K\left(t-t_{i}^{j}\right)-\underset{t_{s}^{j}<t}{\sum }\eta \left(t-t_{s}^{j}\right), \end{array}$$

where *w*_*i*_ is the weight of the synapse established with the *i*-th afferent neuron, and $t_{i}^{j}$ denotes the time of the *j*-th spike from the afferent neuron. $t_{s}^{j}$ denotes the time of *j*-th spike emitted by this postsynaptic neuron. *K*(·) and η(·) characterize the normalized PSP kernel and refractory period, respectively, which are defined as


(2) $$\begin{array}{l} K\left(x\right)=V_{0}\left[\exp\left(-\frac{x}{\tau_{m}}\right)-\exp\left(-\frac{x}{\tau_{s}}\right)\right],\;x>0, \end{array}$$

and


(3) $$\begin{array}{l} \eta \left(x\right)=\vartheta \cdot \exp\left(-\frac{x}{\tau_{m}}\right),\;x>0, \end{array}$$

where τ_*m*_ and τ_*s*_ are the membrane time constant and the synaptic time constant, which together control the shape of the PSP. *V*_0_ is a coefficient that normalizes the PSP. These two kernels only make sense when *x* > 0, since a spike only takes effect at the time after its occurrence.

### 2.2. Voltage slope guided learning

Unlike algorithms that generate an exact desired spike train, VSG aims to generate a desired number of spikes in response to an input pattern. When the actual spike count *N*_*o*_ is more or less than the desired count *N*_*d*_, the network parameters are adjusted:

1. *N*_*o*_ < *N*_*d*_ : When the actual spikes are insufficient, the network parameters are strengthened so that more spikes can be delivered. Thus, the time instant with the largest membrane voltage slope (except the existing spike times) is selected as the critical time t^*^. The membrane voltage *V*(t^*^) at this moment has the strongest upward trend. Adjusting the membrane voltage at this point will be more efficient compared to other locations.
2. *N*_*o*_ > *N*_*d*_ : When more spikes are fired than the expectation, the redundant spikes should be removed by weakening the network parameters. Therefore, the critical moment t^*^ will be selected from the existing spike times. On the contrary, among these moments, the point with the weakest upward trend of membrane voltage crossing the threshold is chosen. Because it can be removed with less effort than other spikes.

As shown in **Figure 3A** (left), the red arrows and green arrows, respectively, represent the critical points if more or less spikes need to be emitted in the case that there are already five output spikes.

For these two cases, we construct error function based on the distance between the critical membrane voltage *V*(t^*^) and its target membrane voltage *V*_*tar*_. In the case of *N*_*o*_ < *N*_*d*_, it is obvious that the target voltage should be equal to the threshold in order to emit more spikes. While in the case of *N*_*o*_ > *N*_*d*_, the critical membrane voltage should be lowered in order to remove the spike, so the target voltage can be set as the resting potential *V*_*rest*_:


(4) $$\begin{array}{l} E=\frac{1}{2}{\left(V\left(t^{*}\right)-V_{tar}\right)}^{2}, \end{array}$$

where


(5) $$V_{tar}=\{\begin{array}{ll} \vartheta , & N_{o}<N_{d},\\ V_{rest}, & N_{o}>N_{d}. \end{array}$$

Then the gradient descent method is applied to obtain the weight updating rule:


(6) $$\begin{array}{l} \text{Δ}\omega_{i}=-\eta \frac{\text{d}E}{\text{d}w_{i}}=-\eta \left(V\left(t^{*}\right)-V_{tar}\right)\frac{\text{d}V\left(t^{*}\right)}{\text{d}w_{i}}, \end{array}$$

η is the learning rate which define the update magnitude of the synaptic weights. In fact, ±η·d*V*/d*w* can also be used directly to enhance/weaken weights during the experiment without considering the error function, which has a learning efficiency similar to Equation (6), as shown in **Figure 6B**.

Without loss of generality, suppose that there is a fully connected network with *L* (*L* ≥ 2) layers. For a neuron *s* in layer *L* (the output layer), if the output spike count is not equal to its desired number, all synaptic weights that contribute to its firing will be adjusted. Assuming that the critical spike time of the neuron is t^*^, and the corresponding membrane voltage is *V*(t^*^). Then according to Equation (6), all we need to do is to calculate d*V*/d*w*:

#### 2.2.1. Output layer

According to Equation (1), *V*(t^*^) is not only affected by the input spikes from the previous layer, but also by the previous spikes $t_{s}^{f}<t^{*}\;\left(f=1,2,...,F\right)$ excited by the neuron itself, therefore,


(7) $$\begin{array}{l} \frac{\text{d}V\left(t^{*}\right)}{\text{d}w_{is}^{L}}=\frac{\partial V\left(t^{*}\right)}{\partial w_{is}^{L}}+\overset{F}{\underset{f=1}{\sum }}\frac{\partial V\left(t^{*}\right)}{\partial t_{s}^{f}}\frac{\partial t_{s}^{f}}{\partial w_{is}^{L}}, \end{array}$$

where $w_{is}^{L}$ is the synaptic weight between *i*-th neuron in the layer *L*−1 and *s*-th neuron in the layer *L*.

From Equation (1), the first term of Equation (7) can be expressed as


(8) $$\begin{array}{l} \frac{\partial V\left(t_{x}\right)}{\partial w_{is}^{L}}=\underset{t_{i}^{j}<t^{x}}{\sum }K\left(t_{x}-t_{i}^{j}\right), \end{array}$$

where $t_{x}\in \left\{t_{s}^{1},t_{s}^{2},\;\cdots \;,t_{s}^{F},t^{*}\right\}$, $t_{i}^{j}$ is the *j*-th spike of the *i*-th neuron in layer *L*−1. While for the second term of Equation (7), we have


(9) $$\begin{array}{l} \frac{\partial V\left(t^{*}\right)}{\partial t_{s}^{f}}=-\frac{\vartheta }{\tau_{m}}\exp\left(-\frac{t^{*}-t_{s}^{f}}{\tau_{m}}\right), \end{array}$$

and


(10) $$\begin{array}{l} \frac{\partial t_{s}^{f}}{\partial w_{is}^{L}}=\frac{\partial t_{s}^{f}}{\partial V\left(t_{s}^{f}\right)}\frac{\partial V\left(t_{s}^{f}\right)}{\partial w_{is}^{L}}, \end{array}$$

where $\partial V\left(t_{s}^{f}\right)/\partial w_{is}^{L}$ can be calculated by Equation (8). Suppose *n*^*l*^ is the number of neurons in the *l*-th layer. Then following the linear hypothesis for the voltage crossing threshold in Bohte et al. (2002) and Yu et al. (2018), we get


(11) $$\begin{array}{l} \frac{\partial t_{s}^{f}}{\partial V\left(t_{s}^{f}\right)}=-{\left(\frac{\partial V\left(t_{s}^{f}\right)}{\partial t_{s}^{f}}\right)}^{-1}=-{\left(\frac{\partial V\left(t\right)}{\partial t}{|}_{t=t_{s}^{f-}}\right)}^{-1}, \end{array}$$

where


(12) $$\begin{array}{l} \frac{\partial V\left(t\right)}{\partial t}=\overset{n^{L}}{\underset{i=1}{\sum }}w_{is}^{L}\underset{t_{i}^{j}<t}{\sum }\kappa \left(t-t_{i}^{j}\right)+\underset{t_{s}^{f}<t}{\sum }\frac{\eta \left(t-t_{s}^{f}\right)}{\tau_{m}}, \end{array}$$

(13) $$\begin{array}{ll} \kappa \left(t-t_{i}^{j}\right)=\frac{\partial K\left(t-t_{i}^{j}\right)}{\partial t} & =\frac{V_{0}}{\tau_{s}}\exp\left(-\frac{t-t_{i}^{j}}{\tau_{s}}\right)\\ \;-\frac{V_{0}}{\tau_{m}}\exp\left(-\frac{t-t_{i}^{j}}{\tau_{m}}\right). \end{array}$$

#### 2.2.2. Hidden layers

Suppose $w_{ij}^{l}$ is the synaptic weight between *i*-th neuron in the layer *l*−1 and *j*-th neuron in the layer *l*. It has an impact on the spike time $t_{j}^{m,l}$, i.e., the *m*-th (*m* = 1, 2, ⋯ ) spike time of the neuron *j* in layer *l*, and then affect the spike time of neurons in all the subsequent layers through $t_{j}^{m,l}$. Therefore, the derivative of *V*(t^*^) with respect to $w_{ih}^{l}\;\left(1\;\leq \;l\leq L-1\right)$ is


(14) $$\begin{array}{l} \frac{\text{d}V\left(t^{*}\right)}{\text{d}w_{ij}^{l}}=\underset{t_{j}^{m,l}<t^{*}}{\sum }\frac{\partial V\left(t^{*}\right)}{\partial t_{j}^{m,l}}\frac{\partial t_{j}^{m,l}}{\partial w_{ij}^{l}}, \end{array}$$

where $\partial t_{j}^{m,l}/\partial w_{ij}^{l}$ can be calculated just like Equation (10). $\partial V\left(t^{*}\right)/\partial t_{j}^{m,l}$, the key term for error propagation between layers, is denoted as $\delta_{j}^{m,l}$.

For 1 ≤ *l*<*L*−1,


(15) $$\begin{array}{l} \delta_{j}^{m,l}≜\frac{\partial V\left(t^{*}\right)}{\partial t_{j}^{m,l}}=\overset{n^{l+1}}{\underset{k=1}{\sum }}\underset{t_{k}^{f,l+1}}{\sum }\frac{\partial V\left(t^{*}\right)}{\partial t_{k}^{f,l+1}}\frac{\partial t_{k}^{f,l+1}}{\partial t_{j}^{m,l}}\\ =\overset{n^{l+1}}{\underset{k=1}{\sum }}\underset{t_{k}^{f,l+1}}{\sum }\delta_{k}^{f,l+1}\cdot \frac{\partial t_{k}^{f,l+1}}{\partial t_{j}^{m,l}},t_{j}^{m,l}<t_{k}^{f,l+1}<t^{*}. \end{array}$$

And for *l* = *L*−1,


(16) $$\begin{array}{l} \delta_{j}^{m,l}=\frac{\partial V\left(t^{*}\right)}{\partial t_{j}^{m,l}}+\underset{t_{j}^{m,l}<t_{s}^{f,L}<t^{*}}{\sum }\frac{\partial V\left(t^{*}\right)}{\partial t_{s}^{f,L}}\frac{\partial t_{s}^{f,L}}{\partial t_{j}^{m,l}}. \end{array}$$

**Figure 2:**
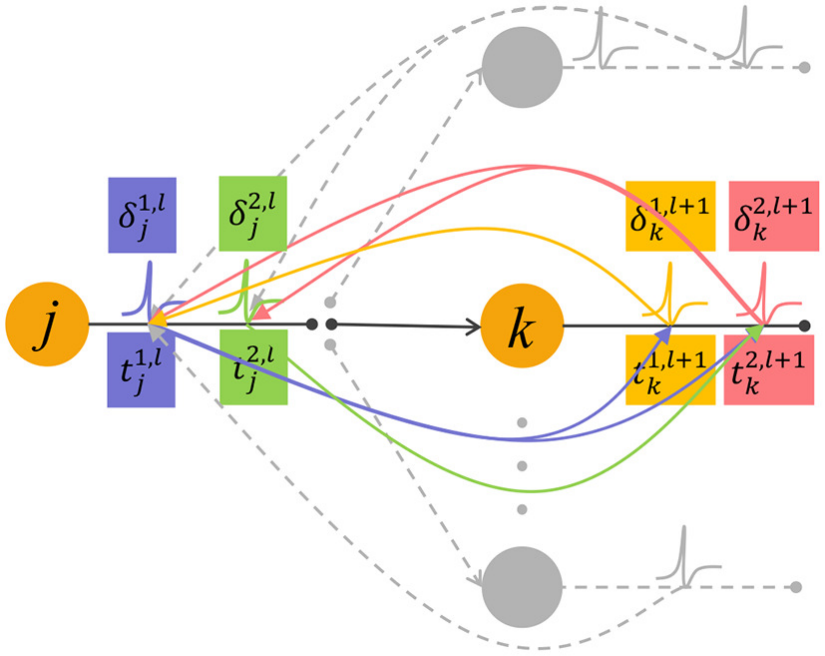
The feedforward propagation of spikes and backpropagation of errors. The neuron *j* in the *l*-th layer emits two spikes $t_{j}^{1,l}$ and $t_{j}^{2,l}$, they affect the generation of spikes in the next layer after them (purple and green curved arrows). The neuron *k* in the layer *l*+1 also emits two spikes denoted as $t_{k}^{1,l+1}$ and $t_{k}^{2,l+1}$. In the feedback process, a spike generated by neuron *k* transmit the error signal δ to input spikes that contribute to it (pink and yellow curved arrows).

Noted that the error backpropagation is performed based on spikes, and Equation (15) involves complex spike time relationships when δ propagate back between adjacent layers. As shown in Figure 2, the spike $t_{j}^{2,l}$ has an effect on the later spike $t_{k}^{2,l+1}$ emitted by the downstream neuron (green arrow), but has no effect on the earlier spike $t_{k}^{1,l+1}$. Therefore, when the error signal $\delta_{k}^{1,l+1}$ corresponds to the spike $t_{k}^{1,l+1}$ is backpropagated, it will only transmit to the earlier spike $t_{j}^{1,l}$ that contribute to it (yellow arrow).

From Equation (1), the first term of Equation (16), i.e., the derivative of the membrane voltage with respect to the input spike coming from its presynaptic neuron is calculated as below


(17) $$\begin{array}{l} \frac{\partial V\left(t^{*}\right)}{\partial t_{j}^{m,L-1}}=-w_{js}^{L}\cdot \kappa \left(t^{*}-t_{j}^{m,L-1}\right), \end{array}$$

and $\partial V\left(t^{*}\right)/\partial t_{s}^{f,L}$ can be calculated by Equation (9). And for 1 ≤ *l* ≤ *L*−1,


(18) $$\begin{array}{l} \begin{array}{c} \frac{\partial t_{k}^{f,l+1}}{\partial t_{j}^{m,l}}=\frac{\partial t_{k}^{f,l+1}}{\partial V\left(t_{k}^{f,l+1}\right)}\frac{\partial V\left(t_{k}^{f,l+1}\right)}{\partial t_{j}^{m,l}}\\ ={\left(\frac{\partial V\left(t_{k}^{f,l+1}\right)}{\partial t_{k}^{f,l+1}}\right)}^{-1}w_{jk}^{l+1}\kappa \left(t_{k}^{f,l+1}-t_{j}^{m,l}\right). \end{array} \end{array}$$

Thereupon, the whole learning process of the VSG is summarized in Algorithm 1.

**Algorithm 1.** Learning algorithm of the VSG.

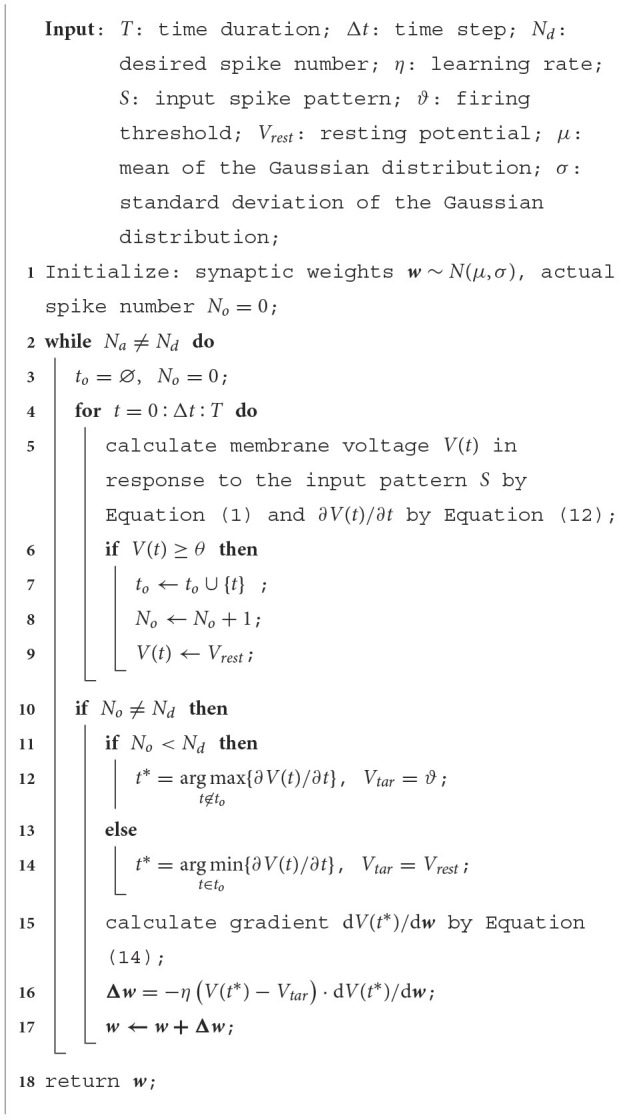

### 2.3. Comparison with other aggregate-label learning algorithms

**Figure 3:**
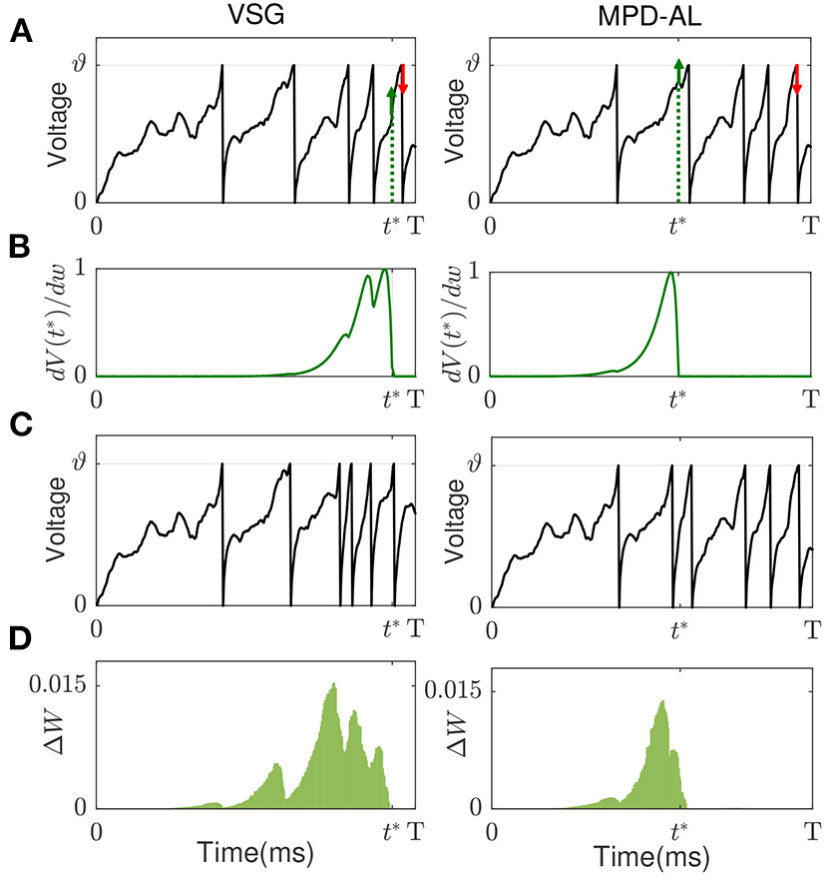
The comparison of learning between VSG and MPD-AL. **(A)** The membrane potential traces before learning. t^*^ indicates the key time point selected when a spike needs to be added. **(B)** Learning curves depicting the spike-timing dependence of the contribution of different synapses to *V*(t^*^). **(C)** The membrane potential traces after learning (with one spike added). **(D)** The amount of weight change before and after learning. For the convenience of observation, each presynaptic neuron sends only one spike, and the synapses in **(B,C)** are arranged in the order of their corresponding spike time.

Existing aggregate-label learning works can be divided into threshold-driven methods, such as MST, TDP1, and membrane voltage-driven methods, such as MPD-AL. The threshold-driven method searches for a critical threshold ϑ^*^ that can increase/decrease the number of spikes by one, then the distance between the critical threshold and the actual firing threshold ϑ is used as the error to update the synaptic weights. However, ϑ^*^ cannot be solved analytically, it can only be obtained by performing dichotomy in the interval where it may appear. Such a search process must be executed for each update iteration, which is quite time-consuming. As for the membrane voltage-driven method MPD-AL, when more spikes are needed, the time of the maximum peak of membrane voltage (below the threshold) is taken as the critical time for enhancing the weights, and when fewer spikes are needed, the last spike time is used as the critical time to weaken the weights, as shown in Figure 3A (right).

Inspired by MPD-AL, we choose the point with the strongest rising trend of membrane voltage at non-spike time and the weakest rising trend of membrane voltage at spike time as the key point for enhancement and weakening, respectively. As shown in Figure 3, taking the addition of a spike as an example, the two algorithms have different choices for t^*^, resulting in different learning curves (Figure 3B), thus adding a new spike in different places (Figure 3C).

**Figure 4:**
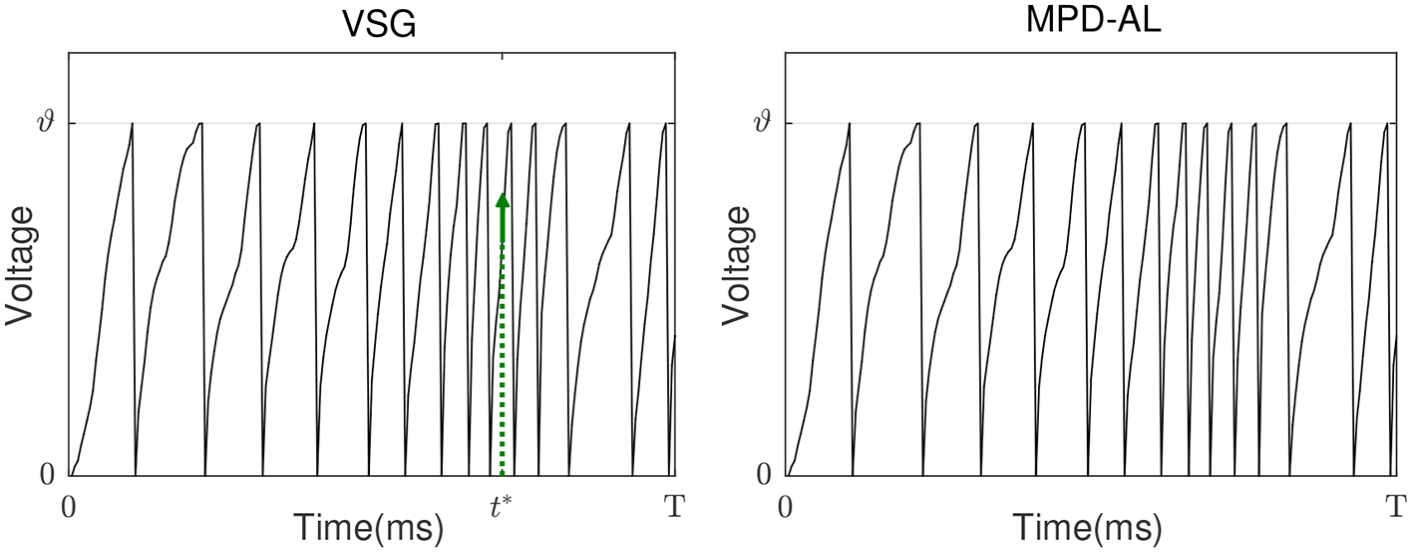
Pain point of MPD-AL. When the membrane voltage rapidly accumulates and frequently emits spikes, there may be no local maximum membrane voltage below the threshold. In this case, MPD-AL cannot find t^*^ if another spike is required **(right)**. However, VSG can find the point where the membrane voltage increases the fastest, namely its t^*^ **(left)**.

Neither VSG nor MPD-AL require the complicated process of finding ϑ^*^, which makes them more efficient than threshold-driven algorithms. However, when a new spike is required, MPD-AL needs to find all local maxima of the membrane voltage below the threshold and then select the largest one. But sometimes such a point does not exist, especially when there are already many spikes, as shown in Figure 4. In this case, MPD-AL can no longer add spikes and the learning stalls. While VSG does not have this problem, because the point with the largest slope must exist, and it is likely to be raised to the threshold quickly, since a large slope means a large upward trend. Similarly, among the firing spike, the point with the lowest slope means that it has less power to cross the threshold, and when a spike needs to be removed, it takes less effort to eliminate it. We will verify the rationality of this selection of adjustment point through experiments in the next section.

On the other hand, VSG seems to be a little more computationally expensive compared to MPD-AL, because it requires additional computation of the time derivative (slope) of membrane voltage. But this calculation can be integrated into the calculation of membrane voltage, since they use exactly the same intermediate variables (Equations 12 and 1). In this way, as shown in **Figure 6A**, it takes almost no more time for VSG to calculate the membrane voltage than MPD-AL, with a total time increase of <0.01 s for 1,000 calculations [the average time for one trial is too small, and the device is Intel(R) Core(TM) i5-8400 CPU @ 2.80, 2.81 GHz]. However, MPD-AL spends about three times as long as VSG in finding the adjustment point. Because it needs to find all the local peak of membrane voltages and then perform the maximum operation, while VSG only needs to perform the maximum operation on the membrane voltage slope. Overall, the computational cost of finding adjustment points for VSG is low.

## 3. Experimental results

Various experiments are carried out to examine the performance of the proposed VSG learning algorithm. We first investigate the learning efficiency of the VSG, and then apply it to learn predictive clues. Several practical classification tasks are performed thereafter to further evaluate its capability.

### 3.1. Learning of desired number of spikes

In this section, we first investigate the ability of a single neuron to learn to deliver a fixed number of spikes through training of VSG algorithm, and then verify the plausibility of its way of finding adjustment points. Finally, it is compared with several competitive aggregation-label learning algorithms to further evaluate its learning efficiency.

**Figure 5:**
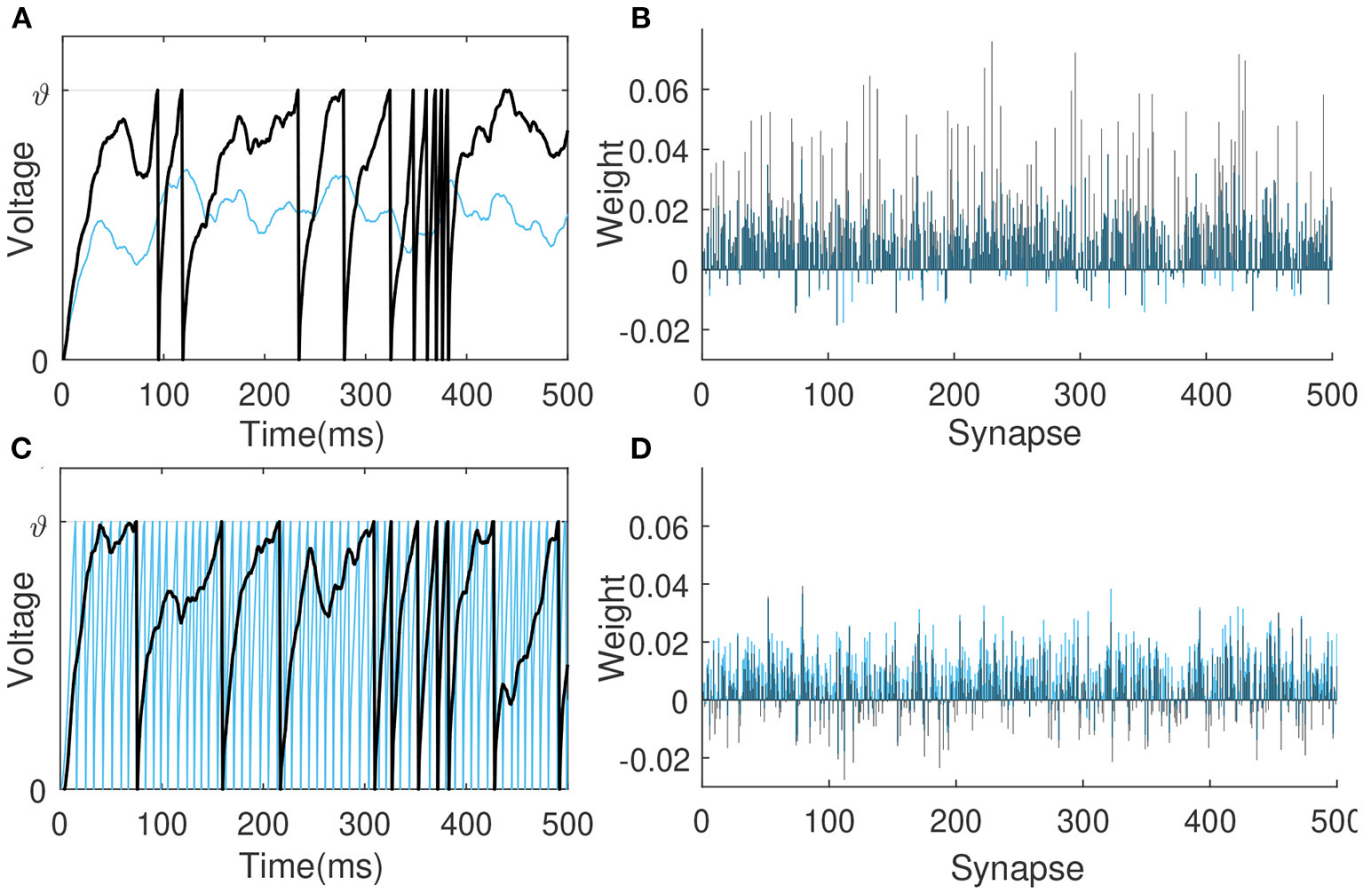
Membrane voltage traces and synaptic weights before (blue) and after learning (black). The learning neuron receives 4 Hz **(A,B)** and 20 Hz **(C,D)** spikes from 500 presynaptic neurons, respectively, and are trained to emit 10 spikes in 500 ms.

In this first experiment, the learning neuron receives spikes from 500 presynaptic neurons and are trained to deliver 10 spikes over a period of 500 ms. To observe the learning under different input conditions, input spikes are generated by the Poisson distribution at 4 and 20 Hz, respectively, while the synaptic weights are initialized by the same Gaussian distribution *N*(0.01, 0.01). Figures 5A,B depicts the membrane voltage traces and synaptic weights of this output neuron before (blue) and after (black) learning when the input spike is 4 Hz. The sparse input caused the neuron not to fire initially, after learning, many synaptic weights are enhanced so that the neuron fires 10 spikes. Figures 5C,D shows the situation of neurons before and after learning when the input spikes is 20 Hz. Before learning, too dense input causes neurons to emit a lot of spikes, and the VSG algorithm weakens the synaptic weights as a whole, so that neurons only emit 10 spikes at the end.

**Figure 6:**
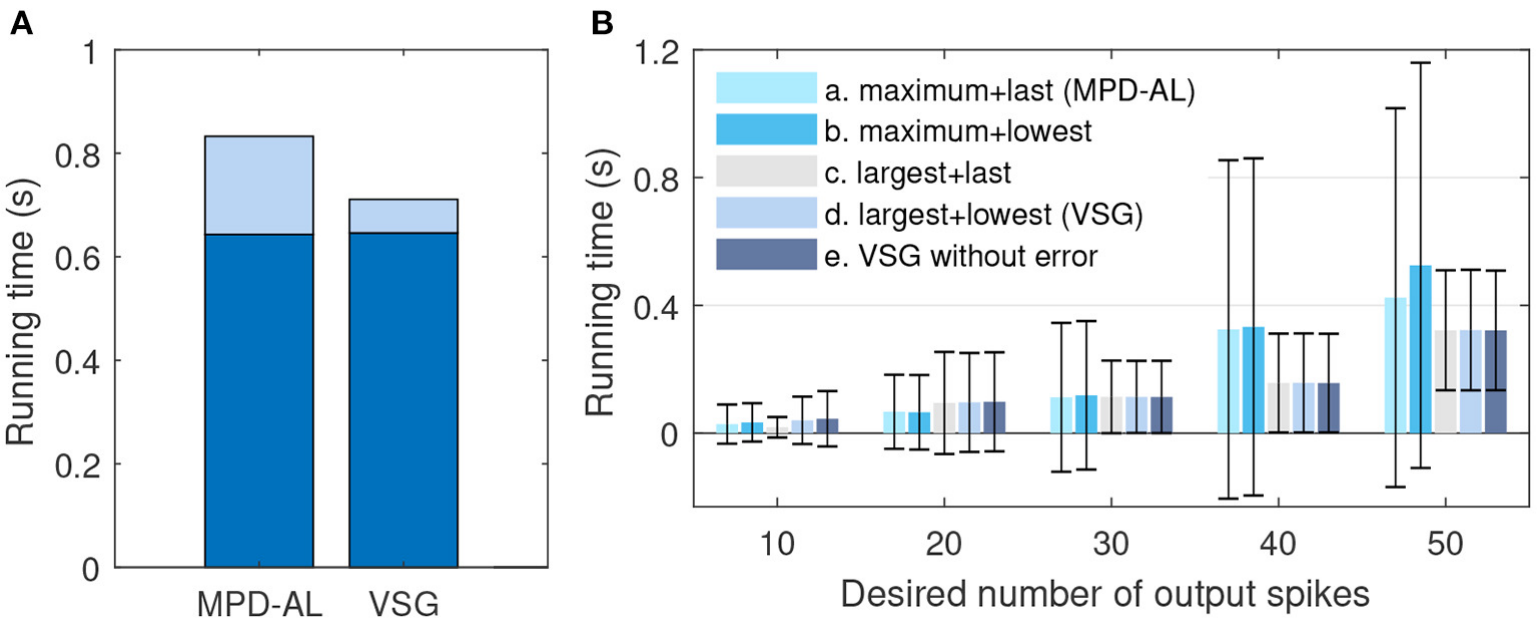
The comparison of efficiency between VSG and MPD-AL. **(A)** The total time to calculate the membrane voltage (dark blue) and find the corresponding adjustment point (light blue) for 1,000 trials. **(B)** The average time required to learn the corresponding number of spikes over 20 trials (up to 2,000 iterations each). For cases where one spike needs to be added and removed, several combinations of methods for finding the adjustment point are tested: (a) maximum peak of subthreshold membrane voltage + last spike (MPD-AL), (b) maximum peak of subthreshold membrane voltage + spike with the lowest slope, (c) non-firing point with the largest slope + last spike, (d) non-firing point with the largest slope + spike with the lowest slope (VSG). In addition, the VSG method without considering the error function (e) is also tested.

Then we verify the rationality of the way the VSG finds adjustment points. We choose different combinations of ways to find adjustment points to test the efficiency of training neurons to emit a specified number of spikes. The firing rate of input is 4–10 Hz, which allows the initial spike count to be more or less than the desired count. Other experimental conditions remain unchanged. The average times over 20 trials for several combinations at each desired spike count are reported. If the neuron does not successfully trigger the corresponding number of spikes until 2,000-th iterations, record the time it took to run 2,000 iterations. As shown in Figure 6B, when the desired number of spikes is small, it is more effective to add a new spike at the maximum peak of the subthreshold membrane voltage. But when the desired number of spikes is large, learning may fail due to the inability to find an adjustable point, and the required time will increase greatly, as shown by the combinations of *a* and *b*. While the method of selecting the point with the largest slope to add a new spike is stable, as shown by the combination of *c*, *d*, and *e*. In addition, by comparing the combination *a* and *b* (or *c* and *d*), it can be found that selecting the spike with the lowest slope or the last spike as the removed spike makes little difference. Therefore, in a nutshell, the way of VSG to find the adjustment point strikes a good balance between efficiency and stability.

**Figure 7:**
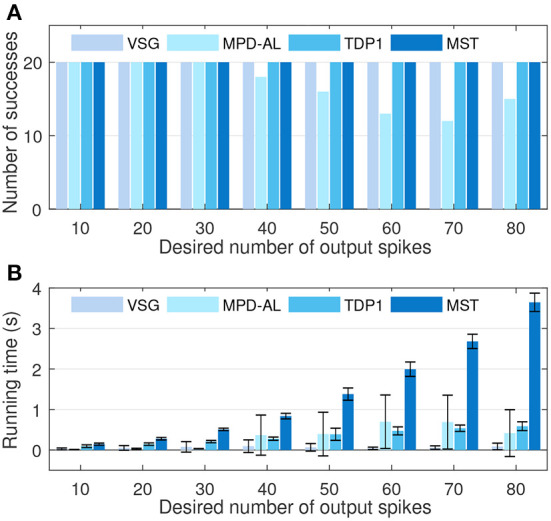
The comparison among VSG, MPD-AL, TDP1, and MST algorithms in terms of learning efficiency. **(A)** The number of successes of learning within 2,000 iterations over 20 trials. **(B)** The required learning time.

Furthermore, we conduct experiments to compare the learning efficiency of VSG and other aggregate-label algorithms. To this end, we test the time required for each algorithm to learn successfully when the desired output count ranges from 10 to 80, with an interval of 10. The firing rate of input is fixed at 4 Hz. Other experimental conditions are the same as above. Figure 7A shows the number of times each algorithm successfully delivered the desired number of spikes over 20 trials. It can be found that when the desired count is greater than or equal to 40, MPD-AL cannot successfully learn every time, because sometimes it cannot find t^*^. While the other three algorithms can learn successfully, even when the number of desired spikes is very large. Figure 7B shows the time required to successfully fire the target number of spikes. The time required for different algorithms almost increases with the increase of the desired spike count, especially MST. The time required for TDP1 is relatively less, but also much more than the proposed algorithm. MPD-AL can learn very quickly only when the required number of spikes is small ( ≤ 30). When the desired spike count is large, the average time it consumes increases significantly due to several failed learning. In short, the learning efficiency of the proposed algorithm is better than other aggregate-label algorithms.

### 3.2. Detection of predictive clues

The task of detecting clues is to simulate the predictive behavior of animals in response to small changes in the environment as they survive in nature. For example, prey may recognize danger by the sound of breaking twigs among many natural noises and flee before predator attacks. Therefore, detecting predictive clues are to identify effective clues hidden within distracting streams of unrelated sensory activity. In addition, there is also a difficulty in how to correlate clues with long-delayed feedback signals, which is called the “temporal-credit assignment problem” (Gütig, 2016). In this section, we will demonstrate the ability of VSG to solve this task.

**Figure 8:**
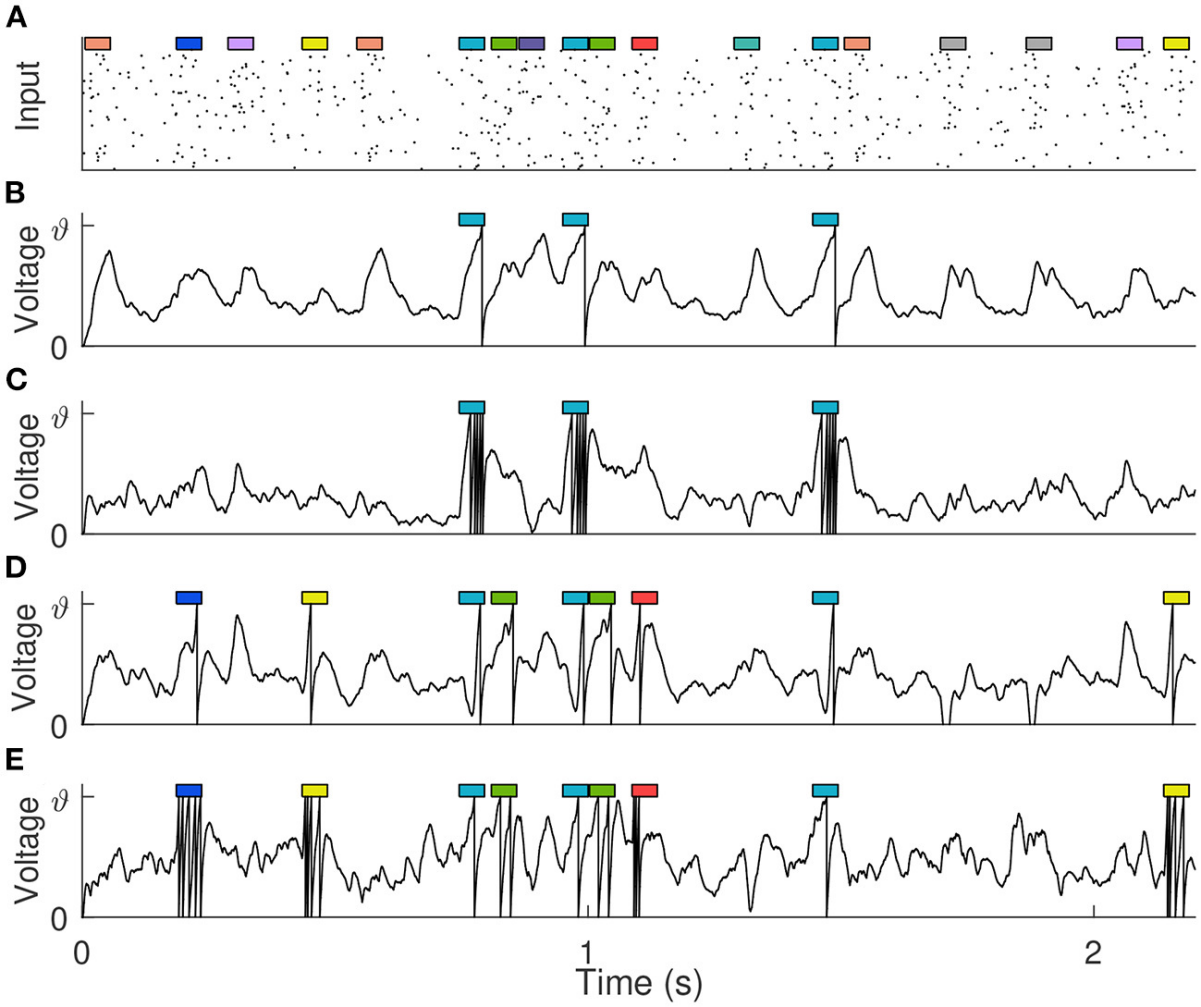
Detection of predictive clues. **(A)** The input spike pattern showing only 100 of the 500 synaptic afferents. 10 different cues (represented by colored rectangles, 50 ms each) are embedded in the background pattern. **(B,C)** The membrane voltage traces of the trained neuron when there is only one kind of effective clue, and it corresponds to 1 and 5 expected output spikes, respectively. **(D,E)** The membrane voltage traces of the trained neuron when there are five kinds of effective clues, and they correspond to {1, 1, 1, 1, 1} and {1, 2, 3, 4, 5} expected output spikes, respectively.

Similar to Gütig (2016) and Zhang et al. (2019), 10 short (50 ms) spiking patterns with firing rate of 4 Hz are generated from 500 afferents to simulate clues, where effective clues and distracting clues are randomly set as required. These clues are then randomly embedded into the background spiking pattern (with duration *T*_*b*_), as shown in Figure 8A, and the number of occurrences of each cue follows a Poisson distribution with mean *P*_*m*_. The firing rate of the background pattern is 0~4 Hz, with an average of 2 Hz, simulating the complex variability of the environment. The single neuron takes the long synthetic spike patterns containing clues and backgrounds as input, and detects effective clues through training, that is, it emits a specified number of spikes at the position where the effective clues appear, while remaining silent where other distracting clues and background patterns appear. During training, a total of 100 training samples are generated for neurons learning, *T*_*b*_ and *P*_*m*_ are set to 500 ms and 0.5, respectively. While in testing phase, in order to make all clues fully exposed, they are set to 2,200 ms and 0.8.

We set up different experiments to detect different kinds of clues. Assuming that *d*_*i*_ spikes are expected to be fired in response to the appearance of clue *i*, and the number of times that clue *i* occurs in a certain sample is *c*_*i*_. Then for this sample, the desired spike count of the learning neuron is $N_{d}=\overset{10}{\underset{1}{\sum }}c_{i}d_{i}$, of which *d*_*i*_ = 0 for distracting clues. During the learning process, if the actual spike count is not equal to *N*_*d*_, the synaptic weight is strengthened or weakened according to the VSG algorithm. We first trained the neuron to detect a single kind of clue, and the remaining nine kinds of clues are distractors. After training, the neuron not only fires the correct number of spikes, but also fires only where the effective clue appears, and remains silent elsewhere. Further, no matter whether *d*_*i*_ corresponding to this effective clue is 1 or 5, the neuron can learn successfully, as shown in Figures 8B,C. Then, we train the neuron to detect five different clues under the conditions that their corresponding spike counts are {1, 1, 1, 1, 1} and {1, 2, 3, 4, 5}, respectively. These involve more complicated temporal-credit assignments. But surprisingly, the neuron can automatically learn effective clues and assign them the corresponding number of spikes based only on the feedback signal of the total number of output spikes, as shown in Figures 8D,E. The experimental results show the capabilities of the VSG algorithm to decompose the delayed output signal and detect effective clues.

### 3.3. Classification of medical datasets

In this section, we test the proposed method on three medical datasets from UCI machine learning repository (Dua and Graff, 2017) and compare with other algorithms.

#### 3.3.1. Data encoding and output decoding

The data encoding refers to encoding real values into spike times. As in Shrestha and Song (2016), Wang et al. (2017), Taherkhani et al. (2018), and Luo et al. (2022), Gaussian receptive field population encoding is used to encode each feature in the original data separately. To encode a certain feature, *K* identically shaped Gaussian functions that overlap each other and cover the interval [*a, b*] are created, where *a, b* are the maximum and minimum values of this feature, respectively. Feeding a real value *x* into these Gaussian functions yields the output value *y*_*i*_ (*i* = 1, 2, ⋯ , *K*), and then inversely mapping *y*_*i*_ to [0, *T*] to get the spike time. *T* is the time window of encoding (in this section, *T* = 100 ms). A large *y*_*i*_ corresponds to an early firing time, a small *y*_*i*_ corresponds to a late firing time, and spikes with time later than 0.9*T* are canceled. Thus, an original sample containing *N* features is encoded as an input pattern containing *KN* neurons, each with at most one spike time. More details about the encoding process can be found in Luo et al. (2022).

Here, for classification tasks, decoding the output refers to determining the category identified by the network from its output. In this section, the number of neurons in the output layer is set equal to the number of categories, and each neuron corresponds to a category. During training, the neuron corresponding to the sample's label is expected to fire *N*_*d*_ (= 5) spikes, while the other output neurons are expected to not fire. In the inference phase, the sample belongs to the class corresponding to the output neuron that emits the most spikes. If no output neuron fires, the sample belongs to the class of neuron with the largest membrane voltage.

#### 3.3.2. Medical datasets and classification results

The Wisconsin Breast Cancer dataset (WBC) contains 699 pieces of data described by 9 features, excluding 16 pieces of data with missing values, 683 samples are used in our experiments. The BUPA Liver Disorders dataset contains 345 samples with six features, and the Pima Diabetes dataset contains 746 samples with eight features. Each of the three datasets has two categories. As in SpikeProp (Shrestha and Song, 2016), SWAT (Wade et al., 2010), SRESN (Dora et al., 2016), and FE-Learn (Luo et al., 2022), we divided the training set and test set in a 1:1 ratio. For data encoding, we use the same number of neurons as in SpikeTemp (Wang et al., 2017) and FE-Learn to encode each feature, shown in Table 1 (# Encoders). A single-layer network and a two-layer network with 360 hidden neurons are used to conduct experiments separately. 20 independent trials are carried out in each experiment, and each trial run 200 epochs. Table 2 reports the mean and standard deviation of the classification accuracy in 20 trials.

**Table 1:** Description of the dataset.

**Dataset**	**WBC**	**Liver disorders**	**Pima diabetes**
No. of instances	683	345	768
No. of categorizes	2	2	2
No. of features	9	6	8
No. of encoders	15	25	10
No. of training	341	172	384
No. of testing	342	173	384

**Table 2:** Comparison of classification performance on medical datasets.

**Dataset**	**Breast cancer**		**Liver disorders**		**Pima diabetes**	
	**Architecture**	**Epochs**	**Architecture**	**Epochs**	**Architecture**	**Epochs**
SpikeProp	55-15-2	1,000	37-15-2	3,000	55-20-2	3,000
SWAT	54-702-2	500	36-468-2	500	54-702-2	500
SRESN	54-(8-12)	306	36-(6-9)	715	54-(9-14)	254
SpikeTemp	135-306	/	150-226	/	80-431	/
Multi DL-ReSuMe	/	100	246-360-2	100	/	100
MPD-AL	135-2	200	150-2	200	80-2	200
FE-Learn	135-2	200	150-2	200	80-2	200
FE-Learn^2^	135-360-2	200	150-360-2	200	80-360-2	200
VSG	135-2	200	150-2	200	Feb-80	200
VSG^2^	135-360-2	200	150-360-2	200	80-360-2	200
	**Train (%)**	**Test (%)**	**Train (%)**	**Test (%)**	**Train (%)**	**Test (%)**
SpikeProp	97.3 ± 0.6	97.2 ± 0.6	71.5 ± 5.2	65.1 ± 4.7	78.6 ± 2.5	76.2 ± 1.8
SWAT	96.5 ± 0.5	95.8 ± 1.0	74.8 ± 2.1	60.9 ± 3.2	77.0 ± 2.1	72.1 ± 1.8
SRESN	97.7 ± 0.6	97.2 ± 0.7	60.4 ± 1.7	59.7 ± 1.7	70.5 ± 2.4	69.9 ± 2.1
SpikeTemp	99.1	98.3	93	58.3	77.5	67.6
Multi DL-ReSuMe	98.2	96.4	69.9	61.8	72.1	70.6
MPD-AL	99.9 ± 0.1	97.2 ± 0.6	92.7 ± 1.8	62.2 ± 3.6	71.4 ± 1.9	69.6 ± 1.3
FE-Learn	94.8 ± 0.9	94.3 ± 1.7	72.2 ± 5.0	61.2 ± 3.6	79.3 ± 1.2	71.2 ± 2.0
FE-Learn^2^	100 ± 0.0	97.5 ± 0.5	96.6 ± 0.7	64.8 ± 2.3	90.6 ± 1.4	72.5 ± 1.5
VSG	99.2 ± 0.5	97.1 ± 0.7	74.7 ± 1.6	63.8 ± 2.0	77.4 ± 1.4	72.3 ± 1.5
VSG^2^	99.3 ± 0.3	97.6 ± 0.6	96.3 ± 8.1	65.1 ± 1.9	91.8 ± 1.8	73.7 ± 1.7

As shown in Table 2, the performance of single-layer VSG is moderate, which is better than that of SWAT, Multilayer DL-ReSuMe (Taherkhani et al., 2018), and single-layer FE-Learn. The two-layer VSG performs better, further outperforming SRESN and two-layer FE-Learn compared to its single-layer counterpart. On the BUPA Liver Disorders dataset, it achieves the highest test accuracy of 65.1% together with SpikeProp, but a smaller standard deviation indicates that it is more stable than SpikeProp. Furthermore, it achieves sub-optimal accuracy on both the WBC and Pima Diabetes datasets. SpikeTemp achieved a state-of-the-art test accuracy of 98.3% on the WBC dataset, but it has a 2:1 ratio of training and test set, meaning it uses more training samples to train the model and fewer test samples to validate, which makes it more advantageous. The accuracy of SpikeProp on the Pima Diabetes dataset is much higher than other methods, but it requires a very large number of training epochs, and it is inferior to the proposed method on the WBC dataset. In conclusion, none of these algorithms can be absolutely dominant, and the performance of the proposed algorithm is relatively excellent.

### 3.4. Classification of speech datasets

In this section, we conduct experiment on speech recognition datasets. As mentioned earlier, VSG can detect useful clues in long spatiotemporal patterns, so it is also suitable for processing signals with rich temporal information like speech signals.

#### 3.4.1. Data encoding and output decoding

**Figure 9:**
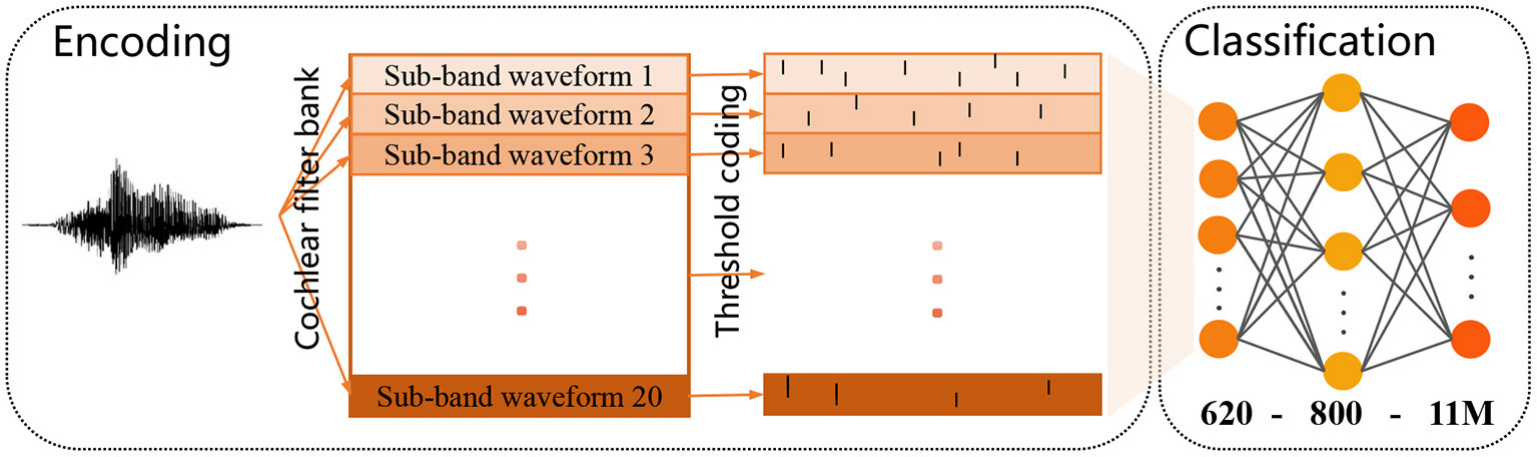
Schematic diagram of the encoding process of a speech sample **(left)** and the applied two-layer classification network **(right)**.

The TIDIGITS corpus (Leonard and Doddington, 1993) is a common dataset widely used for speech recognition (Wu et al., 2018a,b). It consists of 11 isolated spoken digit strings (from “0” to “9,” and “oh”) and speakers from 22 different dialectical regions. 2,464 and 2,486 speech utterances make up the standard training set and testing set. There is already a set of well-established and feasible encoding methods for this dataset: As shown in Figure 9, the raw speech waveform is first filtered by a Constant-Q-Transform (CQT) cochlear filter bank to extract spectral information, where the filter bank consists of 20 cochlear filters from 200 Hz to 8 kHz. Then the threshold coding mechanism (Gütig and Sompolinsky, 2009) is applied to convert the each frequency sub-band into a spike pattern of 31 neurons. Finally, the spike patterns obtained from all frequency bands are spliced into a complete spike pattern of 620 neurons. More details about the encoding process can be found in Pan et al. (2020).

There are also differences among samples of the same category in a dataset, especially for large and complex datasets, for which a fixed number of outputs is unreasonable. Therefore, we adopt the dynamic decoding (DD) strategy (Luo et al., 2019, 2022; Zhang et al., 2019) in this experiment. Instead of specifying a fixed number of output spikes, the dynamic decoding strategy decides whether to add a new spike based on the current sample. Here, we modify the strategy as follows to adapt to the proposed algorithm: If the actual spike count of an output neuron is 1 ≤ *N*_*o*_ < *N*_*d*_, a new spike should be added, but unless the membrane voltage of the selected point reaches a given sub-threshold, i.e., $V\left(t^{*}\right)\geq \vartheta_{s}$, the new point will be discarded and no learning will be performed. This gives the output neuron a degree of freedom to respond to different inputs of the same class.

#### 3.4.2. Network settings and results

The input layer of the network has 620 neurons and is responsible for feeding the encoded spike patterns into the network. The output layer contains 11*M* neurons, of which *M* neurons are a group, corresponding to a class in the dataset. For the group of neurons corresponding to the sample's label, *N*_*d*_ = 5, while the rest of the neurons are expected to not fire (*N*_*d*_ = 0). In the training phase, if the actual number of spikes emitted by a output neuron is not equal to *N*_*d*_, the parameters are adjusted according to the DD strategy (ϑ_*s*_ = 0.8) and the VSG algorithm, where the Adam optimizer (Kingma and Ba, 2015) is also used. During inference, the sample is classified into the class corresponding to the group of neurons with the largest number of output spikes. If all output neurons fail to fire, the sample is considered to belong to the class corresponding to the neuron with the largest membrane voltage. As in the previous section, we use a single-layer network (620 − 11) and a two-layer network with 800 hidden neurons (620 − 800 − 11*M, M* = 1, 10) to conduct experiments separately.

Table 3 shows the highest test accuracies achieved by the proposed method and other baseline methods. A single-layer network trained with VSG can achieve a maximum accuracy of 96.34%. As a single-layer network with only 11 output neurons, it performs well, as the best performing MPD-AL (among single-layer network) has 110 output neurons. In addition, when there is only one set of output neurons (*M* = 1), the two-layer network trained by VSG outperforms the two-layer FE-Learn by a slight advantage. When the number of output neurons is increased (*M* = 10), the performance can be further improved, reaching the highest accuracy of 98.47% as against other baseline methods. However, since the proposed method has only a slight advantage over FE-Learn^2^, it may not have statistical confidence. So we re-executed the proposed algorithm 10 times (500 epochs each) on the two-layer network and reported the average test accuracies (in parentheses). When *M* = 10, the average accuracy is 98.32%, which is also higher than the highest accuracy of FE-Learn^2^. In addition, although the average accuracy when *M* = 1 is only 98.03%, the highest accuracy (98.23%) is higher than that of FE-Learn^2^. We believe that this can demonstrate the superiority of the proposed algorithm.

**Table 3:** Comparison of classification performance on TIDIGITS datasets^*^.

**Model**	**Type**	**Layers**	**Accuracy**
Tavanaei and Maida (2017b)	SNN+SVM	1	91.00%
Tavanaei and Maida (2017a)	Spiking CNN+HMM	3	96.00%
Neil and Liu (2016)	MFCC+RNN	4	96.10%
ETDP (Zhang et al., 2020)	SNN	2	95.80%
MPD-AL (Zhang et al., 2019)	SNN+DD	1	97.52%
FE-Learn (Luo et al., 2019)	SNN+DD	1	96.42%
FE-Learn^2^ (Luo et al., 2022)	SNN+DD	2	98.10%
VSG (*M* = 1)	SNN+DD	1	96.34%
VSG (*M* = 1)	SNN+DD	2	98.23% (98.03%)
VSG (*M* = 10)	SNN+DD	2	98.47% (98.32%)

*DD, dynamic decoding. Values in parentheses are the average of 10 experiments.

## 4. Discussion and conclusion

Temporal-credit assignment problem is a non-trivial problem in machine learning, and the aggregate-label learning algorithm MST is an innovative SNN algorithm to solve this problem. Then TDP1 improves the computational efficiency of MST by modifying the formula for calculating the weight derivative. Subsequently, MDP-AL bypasses the procedure of iteratively finding critical thresholds in the MST and TDP1 by adjusting the weights directly from the membrane voltage, thus greatly reducing the computation time. But there is a drawback in MPD-AL, that is, it may not be able to find the critical time it needs, leading to the failure of learning.

In this paper, we propose to find the potential points for emitting a new spike and the old spike that need to be removed from the time derivative of membrane voltage, avoiding the dilemma of failing to find the adjustment points. Furthermore, on the one hand, the intermediate variables required to calculate this time derivative are also necessary in the calculation of membrane voltage and subsequent weight derivatives, so little additional computation is added. On the other hand, we choose the point with the fastest growth of the time derivative to add the spike, and select the point with the slowest growth of the derivative (among the existing pulses) to remove it, which is experimentally proven to achieve a good balance between efficiency and stability.

A single neuron trained with this algorithm can be used to tackle the challenging temporal-credit assignment problems. Specifically, it can detect valid clues embedded in distracting clues and background spiking activity, deconstruct aggregated delayed feedback signal and then assign them to valid clues. Further, unlike MST, TDP1, and MPD-AL, which is limited to the training of a single neuron or a single-layer network, the proposed algorithm is rooted in multi-layer SNNs for derivation, which further extends its performance. Its application on UCI and speech classification datasets also proves its superiority.

Although the proposed algorithm is simple and efficient, it has drawbacks. Like MPD-AL, when learning predictive clues, if the clues in the training samples are too densely distributed, it will be difficult to learn, which may be an unavoidable problem caused by not calculating the precise critical threshold. In addition, as a multi-layer spike-driven SNN learning algorithm, the proposed learning rule suffers from common problems such as gradient exploding and dead neurons. These all require us to further optimize.

## Data availability statement

The original contributions presented in the study are included in the article/supplementary material, further inquiries can be directed to the corresponding author.

## Author contributions

Both authors listed have made a substantial, direct, and intellectual contribution to the work and approved it for publication.

## Funding

This work was supported in part by the National Natural Science Foundation of China under Grant 82174236 and in part by the Project of Science & Technology Department of Sichuan Province under Grant SYZ202102.

## Conflict of interest

The authors declare that the research was conducted in the absence of any commercial or financial relationships that could be construed as a potential conflict of interest.

## Publisher's note

All claims expressed in this article are solely those of the authors and do not necessarily represent those of their affiliated organizations, or those of the publisher, the editors and the reviewers. Any product that may be evaluated in this article, or claim that may be made by its manufacturer, is not guaranteed or endorsed by the publisher.
